# Thin Film Plastic Antibody-Based Microplate Assay for Human Serum Albumin Determination

**DOI:** 10.3390/polym13111763

**Published:** 2021-05-27

**Authors:** Worachote Boonsriwong, Suticha Chunta, Nonthawat Thepsimanon, Sanita Singsanan, Peter A. Lieberzeit

**Affiliations:** 1 Faculty of Medicine, Bangkok Thonburi University, Bangkok 10170, Thailand; worachota.boo@bkkthon.ac.th; 2 Department of Clinical Chemistry, Faculty of Medical Technology, Prince of Songkla University, Songkhla 90110, Thailand; nonlnwnt145@gmail.com; 3 Department of Medical Technology, Faculty of Allied Health Sciences, Burapha University, Chonburi 20131, Thailand; santa_sing@hotmail.com; 4 Department of Physical Chemistry, Faculty for Chemistry, University of Vienna, 1090 Vienna, Austria; peter.lieberzeit@univie.ac.at

**Keywords:** human serum albumin, thin film MIP-based microplate assay, molecularly imprinted polymer, plastic antibody

## Abstract

Herein we demonstrate molecularly imprinted polymers (MIP) as plastic antibodies for a microplate-based assay. As the most abundant plasma protein, human serum albumin (HSA) was selected as the target analyte model. Thin film MIP was synthesized by the surface molecular imprinting approach using HSA as the template. The optimized polymer consisted of acrylic acid (AA) and *N*-vinylpyrrolidone (VP) in a 2:3 (*w*/*w*) ratio, crosslinked with *N,N′*-(1,2-dihydroxyethylene) bisacrylamide (DHEBA) and then coated on the microplate well. The binding of MIP toward the bound HSA was achieved via the Bradford reaction. The assay revealed a dynamic detection range toward HSA standards in the clinically relevant 1–10 g/dL range, with a 0.01 g/dL detection limit. HSA-MIP showed minimal interference from other serum protein components: γ-globulin had 11% of the HSA response, α-globulin of high-density lipoprotein had 9%, and β-globulin of low-density lipoprotein had 7%. The analytical accuracy of the assay was 89–106% at the 95% confidence interval, with precision at 4–9%. The MIP-coated microplate was stored for 2 months at room temperature without losing its binding ability. The results suggest that the thin film plastic antibody system can be successfully applied to analytical/pseudoimmunological HSA determinations in clinical applications.

## 1. Introduction

A molecularly imprinted polymer (MIP) or plastic antibody is a designed polymer matrix containing selective recognition sites toward a given analyte. MIPs are synthe-sized via molecular imprinting, using functional monomers, crosslinkers, initiators, and a template analyte in a polymerization reaction leading to a highly cross-linked network [1,2,3]. After completion, the template is removed to reveal selective cavities in a polymer matrix that are complementary to the template in size, shape, and positioning of functional groups [4,5]. MIP has become an outstanding selective element, comparable to natural/biological receptors, such as antibodies, enzymes, and DNA. While these natural products offer satisfactory to excellent affinity and specificity, they are often affected by limited ruggedness and long-term stability, poor performance in nonaqueous media, poor reproducibility, and high production costs [6]. Therefore, synthetic receptors, including MIPs, have recently been increasingly studied to overcome these weaknesses. To date, MIP approaches have already resulted in several successful applications covering a wide range of template types, including clinically relevant molecules such as drugs [7], proteins [8], lipids [9,10], lipoproteins [11], viruses [12], bacteria [13], and entire cells [14].

MIP for protein determination is a very fast-growing field, especially for human serum albumin (HSA). It has been frequently chosen as the target analyte model and is also used here because it is the most abundant plasma protein. HSA is synthesized by the liver and is the principal component of blood plasma that maintains colloid osmotic blood pressure [15]. A measurement of HSA concentration is necessary to provide diagnostic/prognostic information of liver functional status [16]. Albumin in the urine is also used as a screening biomarker for renal disease and diabetic mellitus in humans [17]. HSA level is typically determined via one of several alternative quantitative approaches, such as salt fractionation, a dye-binding assay, electrophoresis, or an immunochemical assay [15,17]. The dye-binding technique using bromocresol green (BCG) is the most widely used procedure for quantifying HSA due to its technical simplicity. However, BCG can also aggregate with other serum proteins such as α- and β-globulin [18], and this can interfere with other proteins, leading to overestimates of HSA. Hence, the BCG method should be adopted only for screening tests. Samples with abnormal levels of HSA should be measured using a more specific assay [18]. Method selectivity can be improved by using a specific antibody binding to HSA, as in the gel precipitation technique, electroimmunoassay, radial immunodiffusion, immunonephelometry, and immunoturbidimetry [17,18]. Currently, immunoassays are commonly performed for analytical purposes due to their sensitivity and selectivity. However, there are some limitations from the stability of antibodies that require refrigerated storage/transport conditions. An immunological test is also expensive due to the high cost of antibody production. Therefore, MIPs have attracted increasing attention as potential substitutes for actual antibodies in developing pseudoimmunoassays. Numerous studies have focused on preparing MIPs in nanoparticle format (nanoMIPs) due to their similarity to monoclonal antibodies [19]. NanoMIPs were synthesized by a solid-phase approach that requires immobilizing templates on chemically modified solid phases (e.g., glass beads or silica gel) before adding the prepolymer blend and polymerization. Compared with “simple” synthesis strategies of polymer thin films, creating nanoMIPs is not easy due to the complications and sophistication required in synthesis.

Considering the use in a measurement system, the amount of bound protein on the MIP surface can be determined by indirect and semiquantitative approaches, such as UV absorption spectroscopy, atomic force microscopy (AFM), Fourier transform infrared (FTIR) spectroscopy, and the fluorescent-based binding method [20]. However, these methods are impractical for routine clinical measurements due to requiring sophisticated equipment. Recently, high-throughput MIP-based sensors and assays have been established [21]. Although the sensor is inherently a highly sensitive detector, it is limited by the availability of sensor devices and inconvenient for routine use in a clinical laboratory. Therefore, several examples of the application of a MIP-based pseudo-ELISA assay have been developed and demonstrated for quantification analysis with lab-based spectrophotometers [22]. However, pseudo-ELISA still requires various solutions in each step, e.g., a blocking agent (0.01 M phosphate buffer saline (PBS) containing 0.1% (*w*/*v*) of bovine serum albumin (BSA) and 1% (*v*/*v*) of Tween 20) for preventing the nonspecific binding of the analyte to the well, a horseradish peroxidase (HRP)-conjugate analyte, a substrate solution, and a stopping solution for a color-forming system [23]. Over the past few decades, colorimetric methods have attracted substantial attention due to their simplicity, low cost, and visual response. The most common approach to quantify bound protein is to determine the remaining protein in a solution after protein immobilization using a conventional colorimetric assay. In brief, a known amount of protein is added to bind to a selective material. Then, the amount of residual protein in the supernatant is monitored. Hence, one calculates the amount of bound protein as the difference between the amount added and the residual protein. However, this method cannot be used to determine the amount of the unknown concentration of proteins in a sample. It also requires two measurements, which inherently increases statistical errors.

Given such assay complications in MIP-based pseudo-ELISA and the limitations of the sensor device in a clinical laboratory, the goal of this study lies in combining a straightforward thin film MIP and a microplate colorimetric assay to directly assess protein in a sample quantitatively, using HSA as a model. The colorimetric microplate assay was studied to assess the possibility of replacing antibodies by thin film MIP. This poses substantial challenges in terms of polymer optimization and selectivity. A straightforward dye-binding assay for protein determination, namely the Bradford reaction, was adopted to monitor the bound protein on the MIP surface instead of an enzyme-based colorimetric assay. Currently, there are many available dye-binding assays to monitor protein concentration (i.e., the Bradford method, the Lowry method, and the Biuret method); the Bradford reaction was chosen due to its wide use for protein assessment, leading to a broad dynamic range when quantifying a wide range of proteins. It is also more sensitive and simpler, and it reacts faster than the Lowry and Biuret methods. In addition, this method has been applied successfully to determine conjugated protein on nanoparticles [20], which potentially makes it useful for detecting the protein bound to the MIP.

As an enzyme-free measuring system, this allows for assessing HSA by adding a “one-shot” reagent. This work was also intended to demonstrate the capability of the established material, the thin film MIP-based microplate, to detect HSA in (near) biological matrices. Thin film MIP was integrated as the selective element onto the bottom of a 96-well microplate. The advantages of the microplate technique include the requirement of only small volumes of (bio) chemical reagents, contributing to cost effectiveness. Moreover, a microplate is easier to handle than 96 test tubes in high workload situations. Large batches can be analyzed by preparing MIP thin films in microplate wells before testing, thus shortening the analysis time.

## 2. Materials and Methods

### 2.1. Chemicals and Reagents

Human serum albumin (HSA, lyophilized powder), *N*-vinylpyrrolidone (VP), *N,N′*-(1,2-dihydroxyethylene) bisacrylamide (DHEBA), 2,2-azobis (isobutyronitrile) (AIBN), γ-globulin, and (D+)-glucose monohydrate were purchased from Sigma-Aldrich (Steinheim, Germany). Acrylic acid (AA), magnesium chloride (MgCl_2_), sodium chloride (NaCl), sodium dodecyl sulfate (SDS), potassium chloride (KCl), calcium chloride (CaCl_2_), urea, dimethyl sulfoxide (DMSO), and acetic acid solution were obtained from Merck (Darmstadt, Germany). 4-(2-Hydroxyethyl) piperazine-1-ethanesulfonic acid (HEPES) was obtained from Alfa Aesar (Karlsruhe, Germany). Bradford reagent was purchased from Bio-Rad (Hercules, CA, USA). Low-density lipoprotein (LDL) and high-density lipoprotein (HDL) were isolated from human sera by gradient density ultracentrifugation [24]. All chemicals and reagents were of analytical or the highest synthetic grade commercially available.

### 2.2. Synthesis and Screening of HSA-MIP

**Figure 1:**
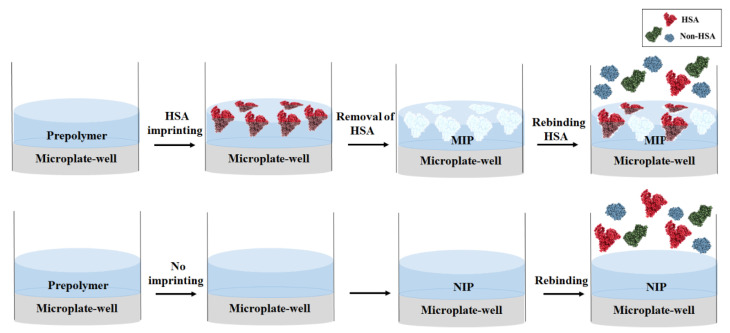
Schematic representation of HSA-MIP-based microplate fabrication.

Polymers were synthesized according to previously published MIP protocols for bovine serum albumin (BSA) [25] due to its closely related properties with HSA. However, they contain different numbers and sequences of amino acids [26]. Moreover, these two albumins also differ in surface hydrophobicity properties, electrophoretic behavior, and thermal and chemical stability. Therefore, polymer optimization was one of the main challenges to determine the optimal condition for MIP synthesis in microplate format. Acrylic acid (AA)/*N*-vinylpyrrolidone (VP) copolymer systems were chosen for HSA-MIP synthesis, because they combine acidic and basic side groups. The strongly polar/dissociable functional groups (i.e., -COO^−^ and -NR_3_^+^) of this system can be expected to undergo interactions with several complementary functional groups of HSA (i.e., -OH, -NH_2_, -COOH, main-chain amide groups, and others) better than other weak/neutral functional monomers. It is necessary to determine the exact mixing ratio of AA and VP to optimize polymer affinities towards HSA. The MIP was prepared by dissolving 15 mg of a functional monomer mixture of AA and VP in varying amounts, 35 mg of *N,N′*-(1,2-dihydroxyethylene) bisacrylamide (DHEBA), and 2.4 mg of 2,2′-azobis (isobutyronitrile) AIBN in 300 μL of DMSO. This solution was prepolymerized under UV at 365 nm and 180 W until approaching the gel point. A polystyrene microplate with 96 flat-type wells was used for easy coating of the thin film polymer. Then, 5 μL of the prepolymer was spin coated at 1500 rpm for 1 min to cover each microplate well. Five microliters of HSA template at a concentration of 20 g/dL in 10 mM PBS, pH 7.4 were then added into the prepolymer film to yield the MIP. Figure 1 sketches the HSA-MIP-based microplate fabrication. Untreated polymer films in select wells serve as nonimprinted polymers (NIP) for nonspecific binding studies. Then, the polymer layer was completely polymerized at 50 °C for 12 h. HSA templates were removed by adding a 10% (*v*/*v*) aqueous solution of acetic acid, followed by a 0.1% (*w*/*v*) sodium dodecyl sulfate (SDS) solution into the microplate wells for 10 min each, and washed with deionized water (DW) 5 times to remove the SDS solution. Many reports indicated that the Bradford assay was affected by the presence of SDS. The SDS solution was removed completely by washing with DW prior to the Bradford assay.

**Figure 2:**
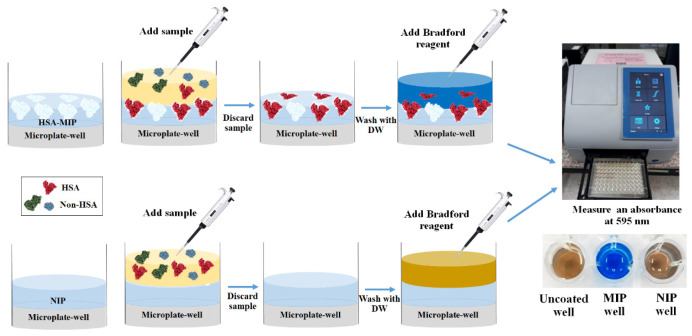
Schematic diagram of HSA determination with the MIP-based microplate assay.

However, the analyte binding events to the MIP-coated microplate did not produce any directly measurable signals. Therefore, a chemical colorimetric assay was combined with the binding assay for indirect detection. To investigate the efficiency of imprinting, the bound HSA on the MIP surface in the microplate was assessed using the Bradford reaction coupled with UV–vis spectrophotometry. The measurement procedure of the bound protein on the polymer surface was modified based on the conventional method, determining the proteins in solution. Briefly, 100 µL of HSA standard at a concentration of 10 g/dL was added to the MIP wells and incubated for 15 min. The HSA solutions were then discarded and the wells were washed with DW three times to remove non-selectively bound molecules. This was followed by adding 200 μL of Bradford reagent and incubating at room temperature (25 °C) for 5 min as shown in Figure 2. The Bradford reagent containing Coomassie Brilliant Blue G-250 dye under acidic conditions can bind to the basic and aromatic amino acid residues of the surface part of the bound HSA on the MIP surface. The reactivities of the dye towards the bound and free protein in solution are different due to their different shapes. Therefore, the two species lead to somewhat different color responses. These differences relate to the number of amino acids that can react with the dye.

The reagent color shifts from 465 nm (reddish brown) to 595 nm (blue) in the presence of proteins. Since the amount of the blue form is proportional to the amount of HSA bound to MIP, one can determine the quantity of bound HSA directly by measuring the absorbance at 595 nm with a Multiskan^TM^ microplate spectrophotometer (Thermo Fisher Scientific, Waltham, MA, USA). In addition, 10 mM PBS without HSA was added to the MIP-coated wells as a blank to calibrate the spectrophotometer reading and correct for absorbance arising from sources other than the analyte, including the remaining SDS. This approach allows for assessing the fraction of accessible binding sites that the HSA molecules occupy for each standard concentration.

### 2.3. Optimization of Template Imprinting

The optimal concentration of the HSA template was evaluated. HSA-MIP-coated wells were prepared by imprinting with 5 µL of different concentrations of HSA at 5–25 g/dL. Then, the imprinting efficiency in terms of the binding efficiency of MIP towards HSA was characterized by using a concentrated HSA solution at 10 g/dL. The concentration of HSA bound to the MIP layer was determined by the Bradford assay as described earlier.

### 2.4. Selectivity Test

The selectivity of the HSA-MIP-based microplate assay was investigated by exposure to other common serum (lipo) proteins at high concentrations that can be found in human serum, namely 5 g/dL of γ-globulin, 1 g/dL of α-globulin (protein part of high-density lipoprotein; HDL), and β-globulin (protein part of low-density lipoprotein; LDL) [26]. “High” refers to clinically high concentrations for each component. In addition, 5 g/dL of BSA was tested to investigate the selectivity of imprinted polymer toward a very closely related structure. Briefly, 100 µL of each protein was added to the MIP wells, incubated for 15 min, washed with DW three times to remove non-selectively bound molecules, and loaded with 200 µL of Bradford reagent. The absorbance of each analyte (*n* = 3) was recorded and compared to the response of HSA.

### 2.5. Thin Film Polymer Characterization

Atomic force microscopy (AFM) and scanning electron microscopy (SEM) served to characterize the polymer surfaces and the thickness of the thin film polymer, respectively. Polymer films were synthesized on clean round glass substrates with a 6 mm diameter, matching the microplate well diameter. Surfaces of the HSA-MIP before and after removing the HSA template and of the NIP were then assessed by AFM using an Asylum Research MFP-3D-BIO in tapping mode with a silicon cantilever (Type Olympus AC160TS) at 1 Hz scan rate. FEI/Apreo SEM was operated at 5 kV to investigate the layer height of each layer on the round glass substrate. All samples were sputter coated with a thin layer of Au before SEM imaging.

### 2.6. Polymer-Based Microplate Assay Characterization

HSA-MIP and NIP were synthesized on the well surfaces as described earlier. Then, the MIP microplate assay was characterized using standards containing different concentrations of HSA (1–20 g/dL) in 10 mM PBS at pH 7.4 to determine the limit of detection (LOD), limit of quantification (LOQ), detection range, and precision. The concentration of HSA bound to the MIP layer was determined by the Bradford assay. The respective absorbance as a function of HSA concentration led to the assay characteristic. In the repeatability test, HSA concentrations of 1 g/dL and 10 g/dL were determined by the MIP-based microplate assay 10 times per day for within-run precision and on 10 different days for between-day precision.

Artificial serum was also prepared to demonstrate the examination of HSA in (near) biological media by adding 40 mg/dL HDL cholesterol (HDL-C), 100 mg/dL LDL cholesterol (LDL-C), 4.5 mM KCl, 5 mM CaCl_2_, 4.7 mM (D+)-glucose monohydrate, 2.5 mM urea, 145 mM NaCl, and 1.6 mM MgCl_2_ in 200 mM HEPES buffer, at pH = 7.4 [27]. Then, artificial serum samples were spiked with different concentrations of HSA to reach final concentrations in the range of 2.5–8.9 g/dL, covering clinically low, normal, and high HSA concentrations. Subsequently, a recovery test was performed to evaluate the accuracy of the assay. The storage stability of the MIP-based microplate was also evaluated by preparing independently MIP-coated wells under the intra-experimental controls and then maintaining the microplates at room temperature (25 °C) for 0–60 days. The MIP binding ability toward the HSA standard solution at concentrations of 5 and 10 g/dL was monitored by measuring the absorbance at 595 nm after the Bradford reaction as described above at 0 days, 15 days, 30 days, 45 days, and 60 days.

An artificial serum sample containing mixed HDL/LDL/γ-globulin at a protein con-centration of 2 g/dL was also prepared. Then, this sample was spiked with different concentrations of HSA to reach final concentrations in the range of 3.0–7.0 g/dL. The HSA concentration of each spiked sample was analyzed using a Pokleritalia 125 Autochem-istry analyzer via the Bromocresol green (BCG) method and the MIP-based assay coupled Bradford reaction.

## 3. Results and Discussion

### 3.1. Optimization of HSA-MIP Synthesis

**Figure 3:**
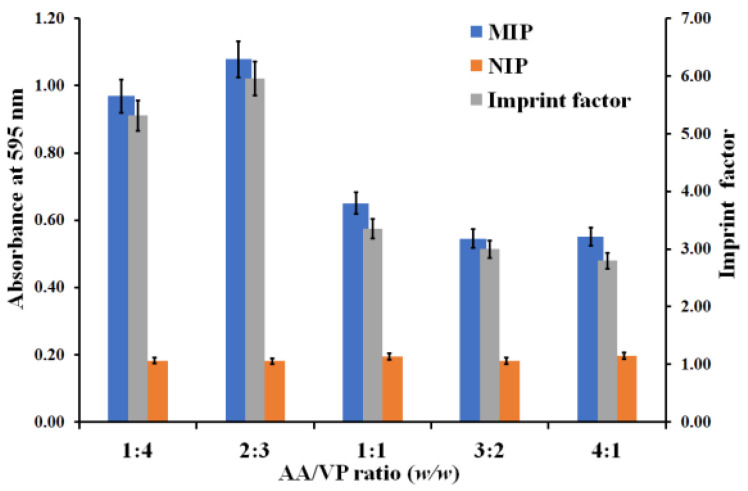
Absorbance of MIP and NIP obtained for varying AA/VP ratios (left-hand axis). The right-hand axis gives the respective imprint factor.

The binding ability of the MIP-coated microplate is the key to successful MIP synthesis. Figure 3 shows the absorbance of rebinding MIP and NIP with 10 g/dL of HSA after the Bradford reaction. The starting point of monomer optimization was an AA/VP ratio of 1:1 (*w*/*w*). The NIP-coated well showed slight absorbance (A_NIP_ = 0.19 ± 0.01) from the reddish brown appearance of the Bradford reagent, which is equivalent to the absorbance of the Bradford reagent in the uncoated polymer well (A = 0.21 ± 0.02). Absorbance of the MIP-coated well (A_MIP_ = 0.65 ± 0.03) exceeded that of the NIP-coated well (A_NIP_ = 0.19 ± 0.01). This corresponded to an imprinting factor (i.e., the absorbance ratio MIP/NIP) of 3.35. The results indicated remaining HSA on the polymer surface and hence corroborated successful imprinting. Then, the optimal ratio of monomers was studied using two groups of polymer compositions, namely the increased AA ratio and the increased VP ratio. For testing the binding ability of the increased AA ratio, MIPs at a ratio of 3:2 (*w*/*w*) and 4:1 (*w*/*w*) yielded lower absorbance (a ratio of 3:2 (*w*/*w*): A_MIP_ = 0.55 ± 0.02, A_NIP_ = 0.18 ± 0.01, a ratio of 4:1 (*w*/*w*): A_MIP_ = 0.56 ± 0.03, A_NIP_ = 0.20 ± 0.01), with respective imprinting factors of 3.05 and 2.75, indicating lower binding affinity of the polymers to HSA. One reason for this is the acidic functionalities on these polymer surfaces: HSA is also an acidic molecule due to its isoelectric point of pI = 5 [28]. Therefore, this impacted interaction with these polymer surfaces. By contrast, MIPs comprising an increased VP ratio with basic functionality of the polymer surface, at a ratio of 2:3 (*w*/*w*) and 1:4 (*w*/*w*), led to higher absorbance (a ratio of 2:3 (*w*/*w*): A_MIP_ = 1.08 ± 0.05, A_NIP_ = 0.18 ± 0.01; a ratio of 1:4 (*w*/*w*): A_MIP_ = 0.97 ± 0.05, A_NIP_ = 0.18 ± 0.01), with imprinting factors of 5.96 and 5.31, respectively. However, AA/VP at a ratio of 2:3 (*w*/*w*) gave the highest absorbance, indicating the best imprinting performance. Therefore, this functional monomer ratio was chosen for carrying out all further experiments.

**Figure 4:**
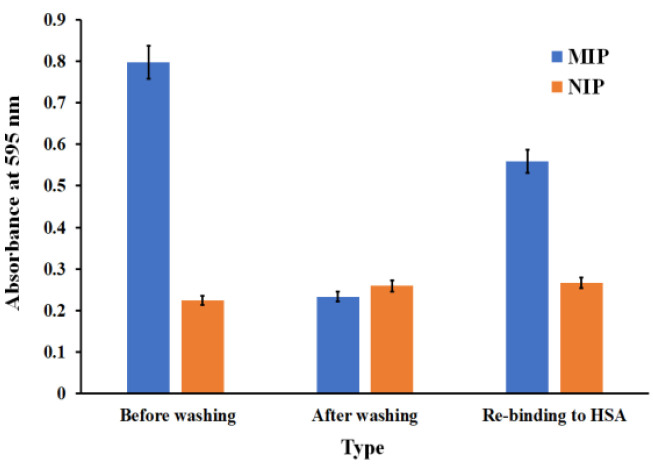
Absorbance of HSA-MIP (AA/VP ratio 2:3 (*w*/*w*)) before removing the template, after removing the template, rebinding with HSA at concentration of 5 g/dL, and the corresponding NIPs.

Figure 4 shows the absorbance at 595 nm for different test samples after the Brad-ford reaction. Results indicated that the NIP-coated well also had slight absorbance (A = 0.22 ± 0.01). By contrast, HSA-MIP before washing had increasing absorbance (A = 0.80 ± 0.04), indicating HSA on the surface. After washing, absorbance decreased (A = 0.24 ± 0.01) to close to that of the NIP signal. This indicates that all bound HSA was removed from the polymer layer by the washing sequence described earlier. Rebinding HSA-MIP with 5 g/dL of HSA generated increased absorbance (A = 0.56 ± 0.03) representing the reuptake of HSA on the HSA-MIP surface, whereas NIP had a slight increase (A = 0.27 ± 0.02).

**Figure 5:**
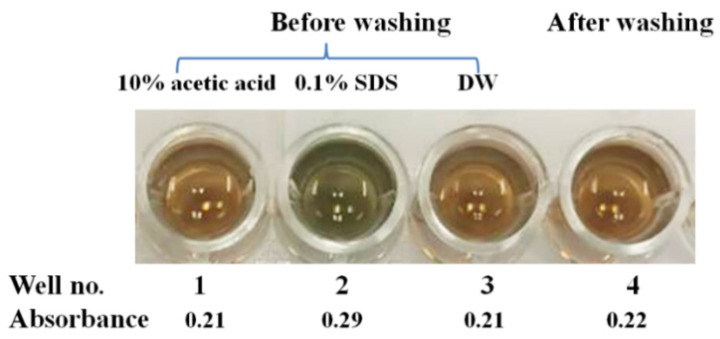
Developed color of the Bradford reagent towards each washing solution on NIP-coated well before washing (well no. 1–3) and after washing (well no. 4).

The increased absorbance of NIP may be caused by the nonspecific binding of protein-dye interaction or by the inferences with chemical-dye interactions. Hence, the reactivity of the Bradford reagent towards each washing solution was tested as shown in Figure 5. Briefly, 200 µL of each washing solution, namely 10% (*v*/*v*) aqueous solution of acetic acid, 0.1% (*w*/*v*) SDS solution, and DW was added into each NIP-coated well for 10 min, and then all solution was discarded. However, each solution still remained on the polymer surface. Then, 200 µL of Bradford reagent was loaded and incubated for 5 min. The absorbance was monitored at 595 nm. The results showed that the SDS-adsorbed NIP well revealed obvious color changes (well no. 2, A = 0.29) compared to other wells (well no. 1 and 3, A = 0.21). This indicates that some SDS remains on the polymer surface and interferes with the Bradford assay. However, in our last washing step, the DW was added to remove the adsorbed 0.1% (*w*/*v*) SDS five times before adding the Bradford reagent. Results showed that the NIP-coated well also had a slight absorbance (well no. 4, A = 0.22) close to that of the only DW-washed well (well no. 3, A = 0.21). This indicated that the 0.1% (*w*/*v*) SDS solution was removed completely prior to the Bradford assay. The results also proved that the washing procedure had been effective, because we did not observe any color changes after that step. Therefore, rebinding the NIP-coated well in Figure 4 revealed slight absorbance changes due to the non-specific binding of the polymer to protein. However, all interference absorbance can be overcome by using the blank well. The MIP-coated well containing the PBS/reagent was used as the blank in the measuring system to correct for absorbance arising from sources other than the analyte.

### 3.2. Optimization of Template Imprinting

**Figure 6:**
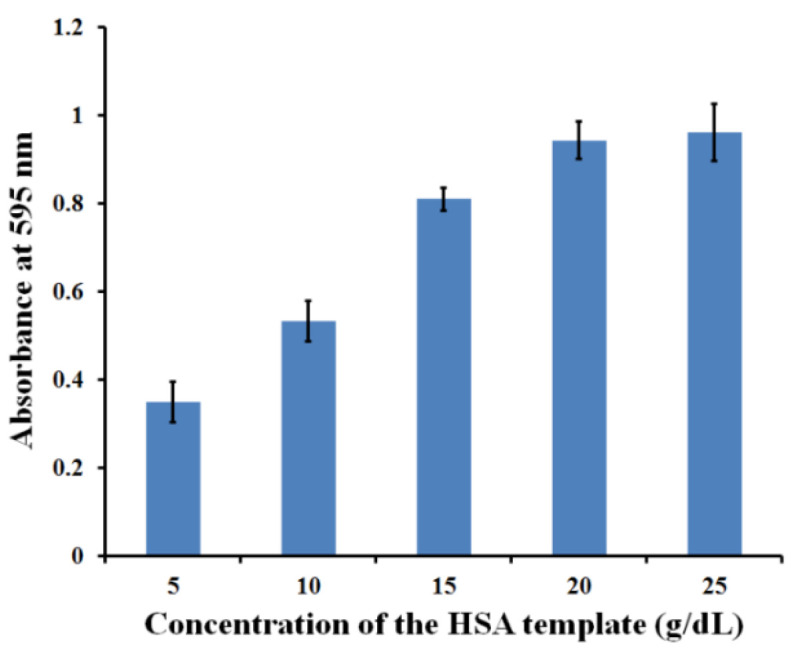
Absorbance of HSA-MIP (AA/VP ratio 2:3 (*w*/*w*)) generated by the various concentra-tions of the HSA template.

Figure 6 shows the absorbance of the bound HSA toward the different MIP surfaces, generated by the various HSA template concentrations. Increasing the HSA concentration led to significantly higher absorbance. The highest absorbance (A = 0.94 ± 0.04) was obtained from the imprinted MIP with HSA at c = 20 g/dL. This absorbance did not significantly change when using higher concentrations of HSA at 25 g/dL (A = 0.96 ± 0.06). Therefore, the HSA template at a concentration of 20 g/dL was chosen as the optimal template concentration for carrying out all further experiments.

### 3.3. Thin Film Polymer Characterization

**Figure 7:**
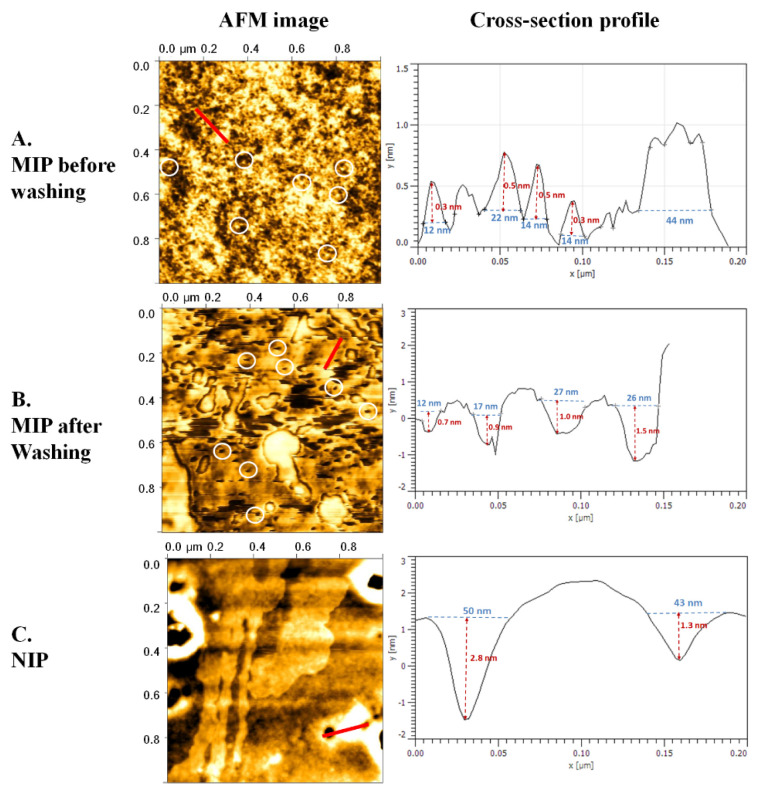
AFM images (Right) and cross-section profiles (Left) of polymers (indicated by red line) before removing HSA (**A**), after removing HSA (**B**), and NIP (**C**). Circles indicate imprinted HSA on polymer (**A**) and imprinted cavities (**B**). Red lines drawn in the right images indicate the region of cross-section analysis.

In the first step of the imprinting experiments, polymer surfaces containing im-printed cavities were verified using AFM. The porosity of the polymer surface can be generated by removing the template molecule after imprinting and by the physical and chemical behavior of the polymer. However, the shape and size of the imprinted cavities should be correlated roughly on the order of magnitude of the imprinted template. Figure 7 shows AFM images and cross-section profiles of MIPs before (Figure 7A) and after (Figure 7B) HSA removal as well as the corresponding NIP (Figure 7C). The surface of the MIP before removing the template was rough and revealed numerous structures that were in the range of 15.5 ± 3.8 nm across and 0.4 ± 0.1 nm high, as shown in Table 1. By contrast, the morphology of HSA-MIP after removing the template was different. The surface was much smoother and displayed numerous pores, both small ones, on average 20.5 ± 7.2 nm across and 7.2 ± 0.3 nm deep, and larger ones. We speculated that these surface features were attributed to the HSA molecules (Figure 7A) and their molecularly imprinted cavities (Figure 7B). The average size of both the observed hills and the pores was somewhat larger than the reported size of albumin (14 × 4 × 4 nm) [29]. However, this increase in size could be due to clustering of the HSA molecules in the prepolymer solution containing DMSO, which is known to induce protein aggregation [30]. Consequently, the formation of large template aggregates composed of two or three molecules of HSA (e.g., size of 44 nm) was observed in a cross-section profile of the template (Figure 7A), leading to large cavities in a cross-section profile of MIP (Figure 7B). By contrast, the NIP surface revealed somewhat large cavities that did not match the HSA in terms of shape and size (Figure 7C). The porosity of the NIP was most probably affected by the physical and chemical behavior of the polymer.

**Figure 8:**
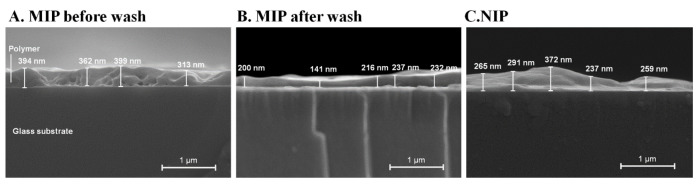
Cross-sectional SEM images of polymers before removing HSA (**A**), after removing HSA (**B**), and NIP (**C**).

Figure 8 displays cross-sectional SEM pictures of polymer-coated glass substrates. The results show that the MIP layer before removing HSA (Figure 8A, 367 ± 40 nm layer height) was the thickest among all layers, due to HSA in the polymer. After washing, the layer height of MIP decreased (Figure 8B, 205 ± 39 nm of layer height), indicating the removal of HSA from the surface. The NIP layer (Figure 8C, 285 ± 52 nm layer height) was thinner than the MIP layer before removing HSA because there was no template for the NIP layers. This strongly supported the successful synthesis of thin film MIP in terms of structure.

### 3.4. Selectivity of HSA-MIP

According to the sensitivity of the Bradford reagent depending on proteins, the reac-tivity of the reagent towards each protein was performed using the gravimetrically prepared 1 g/dL protein solution. Table 2 shows the absorbance of different protein solutions at the same concentration. They revealed a varying color response due to the difference in amino acid sequence, structure, and the presence of certain side chains of each protein. Net color response for HSA was normalized to 1.0 and the net color response of the other proteins was then obtained as a ratio to the response of HSA. To make protein variables comparable to each other in the selectivity test, the absorbance of BSA, γ-globulin, α-globulin, and β-globulin was normalized by the relative factor.

**Figure 9:**
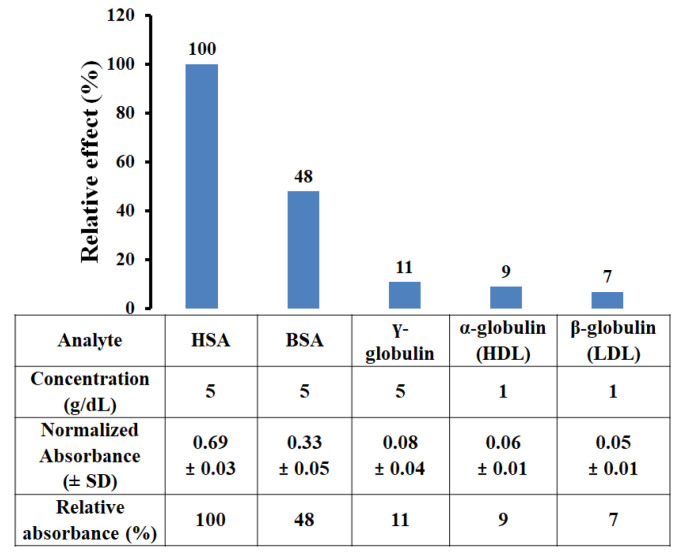
Selectivity of HSA-MIP-based microplate assay in the presence of various suspected in-terferences.

Figure 9 presents the relative effects on the HSA-MIP-based microplate assay of the exposure to different proteins, compared to the HSA response. Adding HSA resulted in absorbance of 0.69 ± 0.03. BSA gave the largest normalized absorbance of the MIP (A = 0.33 ± 0.05) among the suspected protein interferences at the same concentration, because the structures of HSA and BSA are closely related. Generally, HSA and BSA have closely related properties in terms of the size (14 × 4 × 4 nm) and the heart-shaped molecule, but they differ in the number of amino acids (585 amino acids in HSA, 583 in BSA) and in the types of amino acids (145 non-identical amino acids) [31,32]. This led to the highest relative effect of 48%, compared to the HSA response. However, this imprinted polymer still exhibited two times more absorbance with HSA compared to BSA. Despite these similarities, the MIP strongly favored HSA, demonstrating sufficient selectivity of the imprinting approach. BSA is not found in human serum and, therefore, is not significant in clinical practice. All the other (lipo) proteins gave even lower responses, while γ-globulin gave an absorbance of 11% (A = 0.08 ± 0.04) compared to the response to HSA. γ-globulin is a cylindrical particle of 10–20 nm diameter [33] that is somewhat smaller than the HSA cavities (12.8–27.8 nm in diameter). Therefore, γ-globulins were able to enter MIP cavities and generated partial interference in the determination of HSA. Furthermore, HDL contains α-globulin and LDL contains β-globulin, generating 9% (A = 0.06 ± 0.01) and 7% (A = 0.05 ± 0.01) relative responses, respectively. Typically, HDL and LDL particles are much larger (oblate spheroids of 21.5 ± 6.5 nm [34] and 28.9 ± 9.2 nm in diameter [24], respectively) and thus do not fit in the HSA cavities. As discussed earlier, MIP-generated cavities were somewhat larger than the physiological diameter of HSA, potentially due to the clustering of the template molecules. This explains why some HDL and LDL can bind to cavities and be detected in the absorbances.

### 3.5. Polymer-Based Microplate Assay Characterization

**Figure 10:**
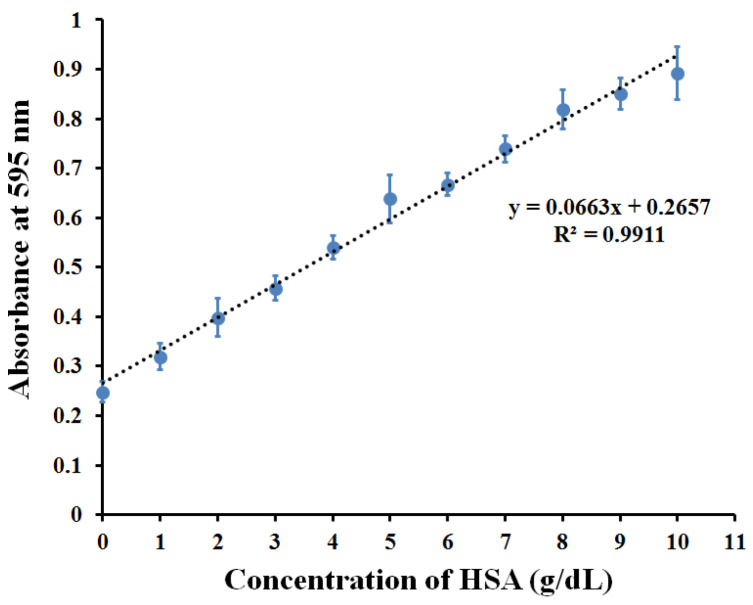
Calibration curve of HSA-MIP-based microplate assay determined with HSA solution.

Figure 10 shows the absorbance generated by the Bradford reaction to selective binding between HSA and MIP in the microplate. Binding HSA-MIP with 1–10 g/dL of HSA generated absorbance proportional to the HSA concentration with a correlation coefficient of R^2^ = 0.9911. Above 10 g/dL (points not shown in the graph), absorbance reached saturation due to the limited number of binding sites on the MIP surface. By evaluating the average absorbance of blank samples (10 mM PBS without HSA) of the MIP (*n* = 3, mean = 0.2509, standard deviation (SD) = 0.0052) plus three and ten times the SD, the estimates of detection and quantification limits are 0.01 and 0.56 g/dL, respectively. Therefore, the detection range of this assay was 0.01–10 g/dL. This covers both normal (reference interval = 3.5–5.0 g/dL) and pathological conditions related to HSA concentration in serum. The MIP microplate assay resulted in a lower detection limit and wider detection range than obtained by the colorimetric bromocresol green assay that was reported in the manufacturer’s brochure (detection limit of 0.2 g/dL, linearity limit of 7.0 g/dL). Therefore, MIP decreased the detection limit of the assay due to preconcentrating the target analytes before colorimetric analysis.

Compared with the previous report of a MIP nanoparticle-based pseudo-ELISA assay for octopamine determination (LOD = 0.017 µg/mL, detection range = 1 nM–0.1 M, measuring time = at least 70 min) [23], our assay provides a higher limit of detection but shorter measuring time (within 20 min) and wider quantitation ranges, and it does not require various solutions used for pseudo-ELISA as described earlier. One reason for the limitation of the LOD is that the assay still contains some intrinsic limitations to prevent lower sensitivity: first, the sensitivity of this assay depends on the limit of detection of the Bradford reaction-based microplate assay that offers the LOD at 0.005 g/dL (reported data in the manufacturer’s brochure). Second, in principle, the number of imprinted cavities of MIP nanoparticles is usually more than the cavities on the thin film, leading to better sensitivity.

Compared to the Bradford interaction towards protein-bound and free forms after adding the standard HSA concentration of 1–10 g/dL, the bound HSA generated absorbance proportional to the concentration between 1–10 g/dL. This indicates that the MIP had a binding ability towards HSA dependent on the amount of HSA in the solution. In contrast to this, the absorbance of the free form reaction (the data not shown) hardly depends on the concentration of HSA, indicating early saturation of the excess amount of HSA in the measuring system. This system behavior correlated to the limitation of the Bradford-based microplate assay, which offers a linearity response at 0.005–0.05 g/dL (the data from the manufacturer’s brochure). Therefore, this system promoted the optimal ratio of protein-dye interaction, improving the Bradford assay’s capability in a wider detection range.

Table 3 demonstrates the recovery rates of the MIP-based microplate assay. These were calculated by comparing the measured and expected concentrations of the spiked HSA in artificial serum. This assay gave recovery rates of 89–106% for low, normal, and high concentrations of 2.5–8.9 g/dL HSA. The accuracy of this assay is inherently acceptable in analytical quality.

The repeatability of the assay was investigated by measuring 10-time repeats of HSA at concentrations of 1 and 10 g/dL. Table 4 shows that the mean and SD values of the microplate assay measurements yielded coefficients of variation at 4–7% and 8–9% for within-run and between-day precision, respectively. This indicates the reliable repeatability of the assay.

**Figure 11:**
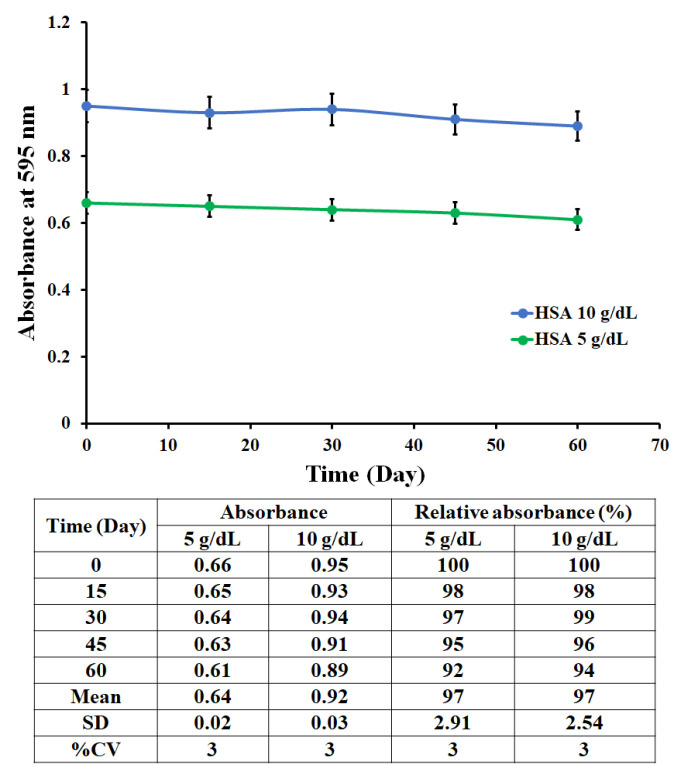
Storage stability test.

Figure 11 shows the absorbance of the MIP-based microplate assay toward HSA solutions at concentrations of 5 and 10 g/dL as functions of storage time up to 60 days at room temperature. These measurements did not show significant changes in absorbance or in assay response, remaining at 92–99% of the first-day measurement. Therefore, long-term storage stability did not require cold chain storage logistics.

**Figure 12:**
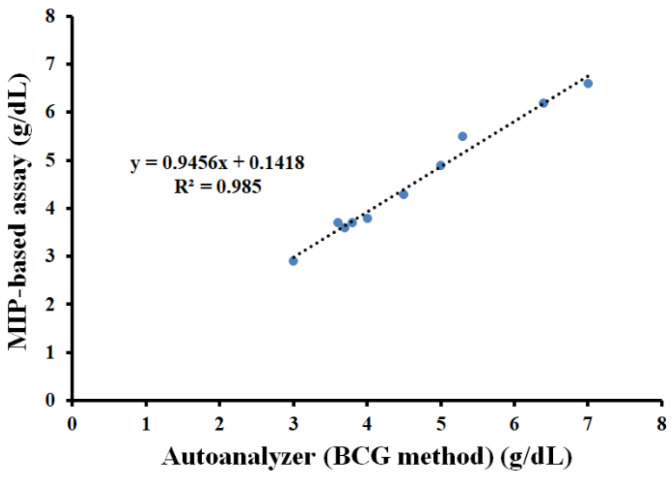
Comparison of HSA-MIP-based assay data (*y*-axis) to the BCG method (*x*-axis) of spiked artificial serum.

Figure 12 shows the linear correlation between the MIP-based assay and the BCG-based autoanalyzer at R^2^ = 0.98, with results similar to the conventional method. However, the MIP-based assay provided a lower limit of detection and a wider dynamic range (0.01–10 g/dL) compared to the BCG method (data from the manufacturer’s brochure; 0.2–7.0 g/dL).

## 4. Conclusions

The molecularly imprinted polymer (MIP)-based microplate assay represents a tech-nologically promising approach to analytical/mimic-immunological assays. Thin film MIP was easily prepared via physical absorption onto the microplate wells to replace traditional antibody coatings. All the results suggest a promising MIP selective binding ability toward HSA with low cross reactivity to other proteins. Our developed assay enables accurate and precise quantification with a wider detection range compared to a conventional colorimetric assay. The detection range also covers the clinically relevant concentrations of HSA. The MIP-based microplate assay was successfully developed and demonstrated for the quantification of HSA. In addition, the platform of thin film MIP microplate combined with the colorimetric assay is promising for further studies on other targets, which requires the selective material to capture the interested target onto the polymer surface before applying colorimetric reagent: changing the template from HSA to other proteins of interest means only minute changes to the assay system. To improve sensitivity, other colorimetric assays can be utilized instead of the Bradford reaction, such as fluorescent-based protein quantification. The appropriate assay should be chosen based on the compatibility of the assay with the samples, the potential interfering substances that affect the assay, the analytical performances, and the incubation time desired.

## Figures and Tables

**Table 1:** Cross-section profiles of AFM images.

Type	MIP before Washing (*n* = 4)		MIP after Washing (*n* = 4)		NIP (*n* = 2)	
	Across (nm)	High (nm)	Across (nm)	Deep (nm)	Across (nm)	Deep (nm)
	12.0	0.3	12.0	0.7	50.0	2.8
	22.0	0.5	17.0	0.9	43.0	1.3
	14.0	0.5	27.0	1.0	-	-
	14.0	0.3	26.0	1.5	-	-
Mean	15.5	0.4	20.5	1.0	46.5	2.1
SD	3.8	0.1	7.2	0.3	4.9	1.1

**Table 2:** Absorbance and relative response of the Bradford reaction towards each protein. Solution at c = 1 g/dL.

Type of Protein	Absorbance	Relative Factor
HSA	1.42 ± 0.03	1.0
BSA	1.29 ± 0.01	0.9
γ-globulin	1.01 ± 0.02	0.7
α-globulin	0.75 ± 0.02	0.5
β-globulin	0.59 ± 0.01	0.4

**Table 3:** Testing of spiked HSA in artificial serum.

Spiked (g/dL)	Measured (*n* = 3)			Recovery (± SD) (%)
	Mean (g/dL)	SD	%CV	
2.5	2.3	0.04	2	92 ± 2
5.5	5.1	0.21	4	93 ± 4
7.4	7.5	0.17	2	101 ± 2
8.5	8.1	0.25	3	95 ± 3
8.9	9.1	0.36	4	102 ± 4

**Table 4:** Precision of HSA-MIP microplate assay.

Type	Concentration of HSA (g/dL)	Mean (g/dL)	SD	%CV
Within-run precision (*n* = 10)	1	0.98	0.07	7
	10	10.16	0.37	4
Between-day precision	1	1.01	0.09	9
(*n* = 10)	10	10.11	0.79	8

## Data Availability

The data presented in this study are available on request from the corresponding author.
